# Legal Notes for Institutional Workers

**Published:** 1916-11-18

**Authors:** 


### LEGAL NOTES FOR INSTITUTIONAL WORKERS.

### Insurance Against Fire of Matrons' and Nurses' Property.

A recent decision of the Court of Session in Scotland deals with a point of much practical interest to all managers or directors of institutions, and indeed to employers generally. A Parish Council took out a policy of insurance over a poorhouse. The policy covered the contents of the building and, *inter alia*, the personal property of the inmates including the servants. The policy was in the Council's name, and the premiums were paid by them. A fire occurred and a considerable amount of property was destroyed, including the personal effects of one of the women superintendents. A claim was made on the insurers which included the value of this personal property. The sum recovered, however, did not amount to the Parish Council's own loss, but out of it the superintendent claimed payment of the value of her property. The Court refused to give effect to her claim. Lord Salvesen said: "I think it is quite an unfounded proposition in law that a person who insures for his own benefit, and incidentally and gratuitously includes in the insurance the property of some other person in whose goods he has no insurable interest, is bound to subordinate his own claim of loss to the claim of the third party."

### Damages for Functional Case.

In two or three recent cases the Court of Appeal have found themselves compelled to consider questions, and, indeed, to use a terminology, very different from those to which they are accustomed. One of these occasions was in the case of *Southampton Gas Company* v. *Stride*, 141 Law Times Jour., 352, where the defendant, an injured workman, showed no objective symptoms to account for his continual inability to walk. The original physical injury had entirely disappeared. His condition was due to loss of will power or defective will power; "functional paraplegia," that is to say, paralysis of the lower limbs, which prevented him from getting about in the way he was able to before the accident. There was no suggestion that he was a malingerer, and that being so, he was, as regards earning his living, held to be in no better case than if his condition was still due to physical damage to his legs. He was accordingly awarded compensation. In so deciding, the Court followed the case of *Eaves* v. *Blaenclydach Colliery Company*, 1909, 2 K.B. 73, a decision which caused some surprise when first given, but which has apparently commended itself to public opinion. The disease there in question was "traumatic neurasthenia," and the effect of the judgment of the Court was to lay down the principle that a mere mental shock sustained through the happening of an accident without any fracture, rupture, abrasion, or otherwise, placed a workman in identically the same position as regards recovering compensation from his employer as a physical injury.

### Importance of a Blood Test.

In actions for damages in respect of negligence, it is a familiar defence that, even though the course alleged to be the right one had been taken, the damage could not have been averted. We notice in an American decision lately reported (*Harvey* v. *Richardson*, Wash. 157, Pac. 674) that the same line of defence has been successfully taken in a case which will interest our readers. It was held that a surgeon who had operated for goitre would not, though the patient died, be refused his remuneration on the ground that he did not take a blood test of the patient, for it had not been established that his failure to do so had injured the patient.

